# On the path to 2025: understanding the Alzheimer’s disease continuum

**DOI:** 10.1186/s13195-017-0283-5

**Published:** 2017-08-09

**Authors:** Paul S. Aisen, Jeffrey Cummings, Clifford R. Jack, John C. Morris, Reisa Sperling, Lutz Frölich, Roy W. Jones, Sherie A. Dowsett, Brandy R. Matthews, Joel Raskin, Philip Scheltens, Bruno Dubois

**Affiliations:** 1 University of Southern California, San Diego, CA USA; 2 Cleveland Clinic Lou Ruvo Center for Brain Health, Las Vegas, NV USA; 3 Department of Geriatric Psychiatry, Central Institute of Mental Health, Medical Faculty Mannheim, University of Heidelberg, Mannheim, Germany; 4 Department of Radiology, Mayo Clinic, Rochester, MN USA; 5 The Research Institute for the Care of Older People (RICE), Royal United Hospital, Bath, UK; 6 Knight Alzheimer Disease Research Center, Washington University School of Medicine, St Louis, MO USA; 7 Center for Alzheimer’s Research and Treatment, Brigham and Women’s Hospital, Harvard Medical School, Boston, MA USA; 8 Eli Lilly and Company, Indianapolis, IN USA; 9 Eli Lilly Canada Inc., Toronto, ON Canada; 10 Department of Neurology & Alzheimer Center, VU University Medical Center, Amsterdam, The Netherlands; 11 Institute for Memory and Alzheimer’s Disease (IM2A) and ICM, Salpêtrière University Hospital, Paris University (UPMC), Paris, France

**Keywords:** Alzheimer’s disease, Amyloid beta, Biomarker, Cognitive impairment, Clinical, Continuum, Dementia, Tau

## Abstract

Basic research advances in recent years have furthered our understanding of the natural history of Alzheimer’s disease (AD). It is now recognized that pathophysiological changes begin many years prior to clinical manifestations of disease and the spectrum of AD spans from clinically asymptomatic to severely impaired. Defining AD purely by its clinical presentation is thus artificial and efforts have been made to recognize the disease based on both clinical and biomarker findings. Advances with biomarkers have also prompted a shift in how the disease is considered as a clinico-pathophysiological entity, with an increasing appreciation that AD should not only be viewed with discrete and defined clinical stages, but as a multifaceted process moving along a seamless continuum. Acknowledging this concept is critical to understanding the development process for disease-modifying therapies, and for initiating effective diagnostic and disease management options. In this article, we discuss the concept of a disease continuum from pathophysiological, biomarker, and clinical perspectives, and highlight the importance of considering AD as a continuum rather than discrete stages. While the pathophysiology of AD has still not been elucidated completely, there is ample evidence to support researchers and clinicians embracing the view of a disease continuum in their study, diagnosis, and management of the disease.

## Background

In the century since Alois Alzheimer discovered Alzheimer’s disease (AD), scientists have made remarkable strides in understanding the illness [1], although it was not until the 1980s that two key molecular culprits in disease pathophysiology, amyloid beta (Aβ) and tau proteins, were identified [2, 3].

Historically, the clinical definition of AD was considered probable because it was based on the systematic exclusion of other potential etiologies in a patient with a dementia syndrome and not on positive proof of AD pathology. Thus, AD could be confirmed only through postmortem findings or, rarely, in life by brain biopsy. Later, AD was defined in a broader sense based on clinical manifestations. With basic research advances in recent years, it is now recognized that, like many chronic diseases, pathophysiological changes begin many years prior to clinical manifestations of disease such that the spectrum of AD spans from clinically asymptomatic to severely impaired. As a result, defining AD purely by its clinical presentation is artificial and efforts have been made to recognize the disease based on both clinical and biomarker findings.

Advances with biomarkers have prompted a shift in how the disease is considered as a clinico-pathophysiological entity, with an increasing appreciation that AD should not be viewed with discrete and defined clinical stages, but as a multifaceted process moving along a continuum. Appreciating this concept is critical to understanding the distinction between AD and AD dementia, in order to initiate effective diagnostic and disease management options and for the development of effective disease-modifying therapies (DMTs).

The aim of this article is to discuss how our understanding of AD pathophysiology and the currently available biomarkers and clinical tools can assist with creating a more uniform approach (in both research and clinical practice) to conceptualizing AD as a continuum. This will help set the stage for the future management of AD and help lay a foundation for the prevention or effective treatment of AD by 2025, the year set by global leaders as the target for finding an effective way to treat or prevent AD [4].

## Understanding the disease continuum

Based on currently available information, AD is best conceptualized as a biological and clinical continuum covering both the preclinical (clinically asymptomatic individuals with evidence of AD pathology) and clinical (symptomatic) phases of AD. In the broadest sense, a continuum is defined as a seamless sequence in which adjacent elements (severities) are not perceptibly different from each other, although the extremes are distinct. In AD, this equates to disease progression from an asymptomatic phase, through a long preclinical period during which pathophysiological changes are reflected by increasing biomarker evidence of disease, to the symptomatic phase, during which biomarker changes continue and symptoms of cognitive and then functional impairment become increasingly evident, with the eventual loss of independence and death. These changes in the individual components of the continuum occur in a sequential but overlapping manner.

## Disease etiology and pathophysiology

The etiology of AD is complex and much remains to be fully elucidated. The close link between genetic mutations and disorders associated with AD (mutations of presenilin 1 (PS1), presenilin 2 (PS2), amyloid beta precursor protein (APP), and Trisomy 21) and the accumulation of Aβ strongly implicates this molecule as a pathological driver in AD, but there is controversy over whether Aβ accumulation alone indicates inevitable progression to AD. Furthermore, evidence indicates that Aβ accumulation alone is probably insufficient to produce symptoms [5–7]. At some point during the disease course, additional factors are involved in determining regional neurodegeneration [8]. Tau pathology has been suggested as a facilitator of the downstream effects of amyloid [9]. Other investigators have proposed that synaptic, mitochondrial, metabolic, inflammatory, neuronal, cytoskeletal, myelin, and other age-related alterations may also play a role in the pathogenesis of AD [10].

Based on our current understanding, histopathological characteristics of AD include 1) accumulation of amyloid plaques—extracellular deposition of Aβ protein, both diffuse plaques of amorphous, primarily nonfibrillar Aβ aggregates and neuritic plaques of fibrillar Aβ arranged in a β-pleated conformation; 2﻿) formation of neurofibrillary tangles (NFTs)—intraneuronal bundles of aggregated tau protein, including hyperphosphorylated tau (p-tau), forming paired helical filaments that aggregate within the neurons to create NFTs, leading to disruption of microtubule function, impaired axonal transport, and synaptic and neuronal injury; and 3) neurodegeneration—progressive loss of neurons or their processes (axons and dendrites) with a corresponding progressive impairment in neuronal function and loss of neurons and synapses (atrophy) [11]. Of note, these features individually are not diagnostic of AD. Several lines of evidence currently suggest that in AD the interplay between Aβ and tau is such that Aβ can drive tau pathology and tau pathology may drive Aβ pathology [12, 13]. Our understanding of the pathophysiology of AD is further complicated by primary age-related tauopathy (PART) [14, 15], a neuropathological condition revealed by tau imaging. PART is characterized by medial–temporal neurofibrillary pathology; the pathology remains localized and there are few or no Aβ deposits.

The transition between healthy aging and preclinical AD is not well defined, at least with our current understanding. This shift is likely subtle and without discernible steps; one can suppose that a combination of genetic and environmental factors plays a role in the process [16]. Genetic factors that may contribute as disease modifiers include the apolipoprotein protein E epsilon 4 (*APOE4*) allele, which conveys an increased risk of disease, and more rapid cognitive decline in the setting of early AD pathology [17]. Other factors that may play a role include cardiovascular risk factors and lifestyle factors such as diet, physical exercise, and cognitive engagement. These lifestyle characteristics influence “cognitive reserve” and onset of objective cognitive decline [18]. The concept of brain or cognitive reserve was originally invoked to provide an explanation for the observation that the extent of AD histopathological changes at autopsy did not always align with the degree of clinical impairment. “Brain reserve” refers to the capacity of the brain to withstand pathological insult, perhaps because of greater synaptic density or a larger number of healthy neurons, such that sufficient neural substrate remains to support normal function. “Cognitive reserve” is thought to represent the ability to engage alternate brain networks or cognitive strategies to cope with the effects of encroaching pathology. While cognitive reserve may help delay the onset of clinical symptoms, once symptoms do emerge the rate of impairment may be greater [18] (i.e., there is a steeper trajectory for clinical impairment) because, while clinical symptoms are delayed, pathological changes progress.

The challenges in elucidating when pathophysiological changes start in the brain make it difficult to define a time of disease onset. Findings from epidemiologic studies suggest that midlife or earlier exposures (e.g., hypertension, smoking, diabetes, and obesity) increase the risk for subsequent clinically diagnosed AD dementia [19–21]. Available data are largely cross-sectional, although long-term population studies also provide evidence to suggest that late-life dementia may be linked to exposures occurring in early and midlife; these studies have not focused on AD specifically, and biomarkers were not considered. Recently initiated prospective cohort studies such as the PREVENT Project [22] will yield important information on the interplay between risk factors, biological and clinical changes, and the sequence of disease processes. Based on what we know today about AD pathophysiology and the sensitivity of currently available biomarkers, the starting point of disease is generally defined as when there is specific biomarker evidence of disease, more specifically the demonstration of Aβ accumulation, as revealed by positron emission tomography (PET) or cerebrospinal fluid (CSF) analysis (Table 1).

**Table 1:** Biomarkers currently in use in the AD field

Biomarker	Findings in AD – other relevant notes
CSF analysis	
Aβ_1–42_	Reduced concentration – Measures soluble forms of Aβ – Result of equilibrium shifts due to deposition/aggregation of Aβ_1–42_ in brain parenchyma or decreased production of Aβ_1–42_ – Level inversely reflects brain Aβ burden
t-tau, p-tau	Increased concentrations – t-tau reflects the intensity of neuronal degeneration; it is elevated in other conditions such as head trauma, CJD, and stroke and therefore not specific for AD – p-tau is a marker of the abnormal pathophysiology associated with neurofibrillary tangle pathology (hyperphosphorylation) in the brain. It is fairly specific for AD and is not elevated in primary tauopathies, head injury, or stroke
PET scan^a^	
Amyloid PET	Retention of amyloid tracer – Amyloid tracers include ^11^C-PiB, florbetapir (AV-45), 91 flutemetamol (^18^F-PiB derivative), florbetaben (AV-1), and AZD4694 – Identifies fibrillar Aβ and provides information about extent of Aβ plaque burden in brain
FDG PET	Evidence of reduced temporo-parietal glucose metabolism – Flurodeoxyglucose (^18^F) tracer – Sensitive marker of synaptic dysfunction
Tau PET	Retention of tau tracer – Tau tracers include flortaucipir (18 F-AV1451). Others are in development
MRI	
fMRI	Measure of function – Detects differences in BOLD signals over time and space – Task-associated/based fMRI measures spatio-temporal changes in BOLD signal associated with administration of a task during the scan – Resting state fMRI measures spatio-temporal correlations in intrinsic or spontaneous fluctuation of BOLD signal – Used to study functional networks (e.g., the default mode network)
vMRI	Volume or cortical thickness reduced – Provides a measure of volume of whole brain, specific anatomical regions, or cortical thickness – Demonstrates medial temporal atrophy and, more specifically, hippocampal atrophy; hippocampal volume is reduced in many conditions, including old age, and several neurodegenerative disorders as well as nonneurodegenerative disorders (e.g., diabetes, sleep apnea, bipolar disorder)

*Aβ* amyloid beta, *AD* Alzheimer’s disease, *CSF* cerebrospinal fluid, *t-tau* total tau, *p-tau* phosphorylated tau, *FDG* flurodeoxyglucose, *PiB* Pittsburgh compound B, *PET* positron emission tomography, *MRI* magnetic resonance imaging, *fMRI* functional MRI, *vMRI* volumentric MRI, *BOLD* blood oxygen level dependent, *CJD* Creutzfeldt-Jakob disease

^a^Uses specific ligands to detect AD pathophysiology in the brain

## Biomarker and clinical findings along the continuum

The AD continuum is composed of multiple interconnected components (pathophysiological processes, biomarker findings, and clinical symptoms), each occurring on its own trajectory, with individual trajectories generally parallel to each other but with some temporal offsets. The trajectories are influenced by modulating factors and, for both biomarkers and clinical symptoms, are dependent upon the sensitivity of the measurement.

### The preclinical phase of AD

**Fig. 1:**
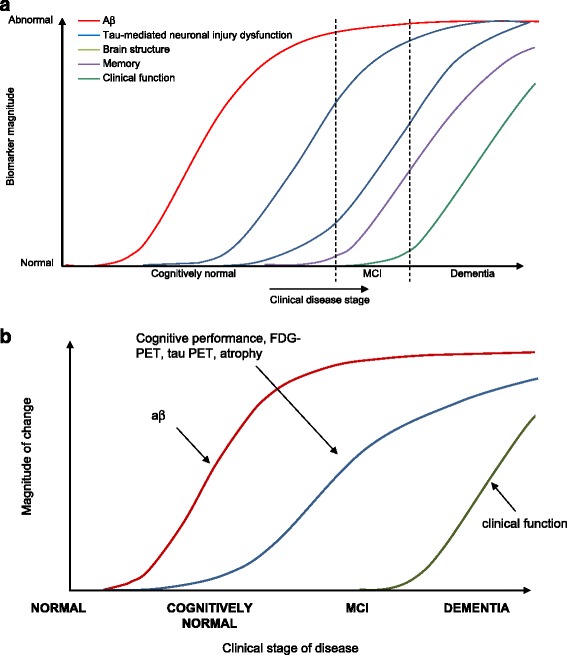
Change in biomarkers over time. **a** Sequential change in measures of AD. Reproduced with permission from [24]. **b** Modified graph showing that amyloid accumulation (measured as low CSF Aβ or elevated amyloid PET standard uptake value ratio) occurs first and functional decline occurs late in the continuum of AD (as before), but cognitive performance, FDG-PET, tau PET, and MRI atrophy change along a common, gradually steepening curve. *Aβ* amyloid beta, *FDG* flurodeoxyglucose, *PET* positron emission tomography, *MCI* mild cognitive impairment due to Alzheimer’s disease

Exploring the continuum through the preclinical phase requires the use of biomarkers because individuals are apparently clinically normal. A number of diagnostic and progression markers have been described (Table 1), with the distinction being that diagnostic biomarkers are direct surrogates of brain AD lesions (amyloidosis or tauopathy) and therefore indicative of the presence of the disease, irrespective of stage, while progression markers identify downstream changes (metabolic changes, neuronal loss with atrophy) indicative of disease progression but not necessarily specific to AD [23]. Biomarker trajectories paralleling the hypothetical pathophysiological sequence of AD and specific biomarker changes over time were described by Jack et al. [24, 25] (Fig. 1).

The model presents the temporal evolution of five well-established AD biomarkers that provide a measure of brain Aβ deposition, tau, or neurodegeneration both in relation to each other and to the onset and progression of clinical symptoms. The model assumes that each biomarker follows a nonlinear temporal course, which is hypothesized to be sigmoid shaped, and that the maximum rate of change moves sequentially from one biomarker to the next. While there is some individual variation, changes in markers of Aβ deposition generally precede those of tau and neurodegeneration. The model not only describes biomarker trajectories in the preclinical phase, but also in the clinical phase, when symptoms become evident. Support for this hypothetical model has been provided by cross-sectional biomarker data across the preclinical/clinical continuum [26–28].

### The transition to the clinical phase of AD

Whether there is a specific threshold or regional distribution of AD pathology and/or a specific combination of biomarker abnormalities that will best predict the emergence of clinical symptoms remains unclear. Cognitive decline will likely occur only where there is Aβ accumulation plus other changes—synaptic dysfunction and/or paired helical filament tangle formation, neurodegeneration, and neuronal loss [29, 30].

The time between Aβ accumulation and clinical symptoms remains to be quantified, but current theories suggest that the onset of cognitive decline lags by at least 15 years [26, 31–33]. There are interindividual differences and some older individuals with preclinical evidence of pathophysiological changes may not become symptomatic during their lifetime, potentially the result of a more slowly progressing disease or death due to a competitive mortality. These interindividual differences are attributable to both environmental and genetic factors, including brain reserve, cognitive reserve, and genetic polymorphisms, as well as coexisting pathologies (age-related brain diseases) and medical comorbidities. As an example of the role of genetic factors, one allele of *APOE4* shifts the risk curve for the disease to 5 years earlier, two copies of *APOE4* shift it 10 years earlier, and one copy of the *APOE2* allele shifts it 5 years later [34, 35].

The distinction between preclinical (asymptomatic) and early clinical (symptomatic) disease is subtle, and clinical manifestations of AD do not become apparent abruptly. Individuals with preclinical AD exhibit longitudinal decline on cognitive assessments even in the absence of clinically significant symptoms [17, 36]. Some individuals are aware of subtle changes in cognitive function before they are detectable using currently available measures of episodic memory, psychomotor speed, verbal fluency, and concept formation [37–39].

### Advancing along the clinical continuum

The clinical continuum includes trajectories for both cognition and function, each of which could be divided into further individual trajectories—for example, cognition includes trajectories of episodic memory, executive function, and verbal fluency; and function includes trajectories of both basic and complex activities of daily living (ADLs). Based on our current ability to detect deficits, cognition and function appear to decline on temporally offset trajectories and cognitive impairment precedes and predicts functional impairment [40].

While the concept of a continuum is biologically more appropriate for characterization of the course of AD, some degree of staging has been helpful for clinical purposes; for example, assessing public health impact, clinical research purposes (trial population selection), standardized patient assessment and management in clinical practice, and health service utilization studies. Traditionally, the two key clinical stages have been considered mild cognitive impairment (MCI) due to AD, or prodromal AD, and AD dementia, with dementia further divided into mild, moderate, and severe [41–43]. However, terminology such as “mild AD” and “moderate AD” is inaccurate—by the time an individual has mild or moderate dementia, they are far along the continuum and the disease has been present for many years, highlighting the important distinction between the syndromic and neuropathological diagnoses. In addition, while clinical staging nomenclature infers a clear distinction between the various stages, in reality the process progresses in a more continuous manner.

Whether or not one embraces clinical staging and the distinction between MCI and dementia, what is clear is that advancing along the clinical continuum there is progressive impairment of cognitive abilities and function. Cognitive deficits are often first apparent in episodic memory, and there is a specific profile of memory deficits, the amnestic syndrome, of “hippocampal type” [23, 43], characterized by diminished free and cued recall ability. This profile has been shown to be highly predictive for the presence of AD pathology [44]. Episodic memory loss is followed by or accompanies executive dysfunction (e.g., impaired planning and anticipation or failures on tests such as dual tasking and response inhibition), and language and recognition difficulties. Functional impairment is usually first apparent as subtle deficits in complex ADLs, such as problems with medication intake, telephone use, financial decisions, keeping appointments, and using everyday technology [45]. Impairment in basic ADL function (such as eating, dressing, and toileting) is generally not apparent until further along the clinical continuum. In a recurring theme, there are individual differences in rate of cognitive and functional decline, and not all individuals will progress to AD dementia or through the various AD dementia severities during their lifetime.

## Clinical implications of the continuum

For the clinician, applying the continuum concept of AD raises several points of importance for counseling and prognostic discussions. AD can be diagnosed without dementia; an understanding of the biomarker changes is equally important to understanding clinical manifestations; and an appreciation for the temporal course of AD from preclinical biomarker evidence of disease through the presence of clinical symptoms is critical for effective disease diagnosis and management.

As 2025 approaches, there are other considerations concerning the continuum concept. There is currently no cure available but if forthcoming new treatments include DMTs that may have more impact if initiated earlier in the disease process, then earlier detection of disease is necessary. For more effective disease management, we also need to predict the future clinical course of disease more accurately. To achieve these goals, more research is needed to define biomarker profiles that best predict progression from the preclinical to the clinical stage and biomarker and/or cognitive profiles that best predict progression and rate of progression along the clinical course.

## Role of biomarker assessment

While we know that pathological changes begin long before symptoms appear, determining whether biomarker evidence of pathophysiological changes in the preclinical stage implies definite progression to clinical disease during an individual’s lifetime is difficult. Individual biomarkers do not provide definitive prognostic information. Recently, there have been efforts to improve diagnostic accuracy and ability to predict those at risk for clinical symptoms by considering a combination of biomarker findings. Jack et al. [46] proposed that diagnosis should be based on both the presence and absence of seven biomarkers in three categories (amyloid, tau, and neurodegeneration (A/T/N)). Dubois et al. [47] proposed that diagnosis be based on both Aβ and tau pathology. Using these criteria, the authors went further to differentiate between a “state” and a “stage”. In simple terms, a state is considered asymptomatic at risk of AD (cognitively normal and amyloid or tau positive but not both) or AD (amyloid and tau positive), while a stage refers to the degree of disease progression within a given state (e.g., clinical AD, preclinical AD, MCI due to AD or prodromal AD, dementia due to AD). Conceptualizing AD as a continuum may favor describing the state as dichotomous and the stage as continuous.

Only a few years ago, diagnosis supported by biomarker findings (Table 1) was considered appropriate only for research-related purposes [41]; however, there is increasing effort to integrate biomarkers into clinical decision-making, with several PET tracers having received regulatory approval and CSF markers being measured with higher precision using advanced automated systems. CSF Aβ_42_, total tau (t-tau), and phosphorylated tau (p-tau) biomarkers and amyloid PET have been the most widely studied [48, 49]. More recently, tau PET has shown promise as a fairly specific marker of tau deposits characteristic of AD [30, 50].

Biomarkers are now a key component of AD clinical trials, playing a central role in selecting individuals for whom the study treatment is most likely to be effective and providing objective evidence of target engagement and disease-modifying effects. Integration of biomarker assessment into more widespread clinical use would help with earlier and more accurate diagnosis. For example, approximately 25% of individuals referred for clinical trial participation with a clinical diagnosis of mild AD dementia have been shown to be Aβ-negative [51], an observation inconsistent with the clinico-biological concept of AD, and this may be even higher in MCI due to AD [52]. Biomarker assessment would also assist with creation of uniform disease staging criteria for use in clinical research and practice, to enhance communication between research and practice, and help with transition of AD treatments from research to regulatory review to clinical practice. However, the clinical care environment has not yet evolved adequately for this to occur. In part, this is due to challenges with cost, standardization, accessibility, and incorporation of biomarker findings into patient treatment plans, but also, as mentioned previously, currently available biomarkers have limited ability to predict the clinical disease course. For clinical practice, we need simpler, less invasive, and more affordable biomarkers, as well as biomarkers that relate to other aspects of the disease.

## Role of clinical assessment

Clinical assessment still provides the central approach to patient evaluation and should incorporate history taking from both patients and knowledgeable informants, supplemented by the use of cognitive and functional assessment tools. For detection of AD in the primary care setting, brief cognitive screening tools with adequate sensitivity may be helpful, although careful history taking from the individual and the family is essential. Tools that may be considered include questionnaires that probe for early change (e.g., Eight-item Interview to Differentiate Aging and Dementia (AD8), Cognitive Function Instrument (CFI)), brief global cognitive screens (e.g., MMSE), and more specific tests of episodic memory impairment indicative of hippocampal dysfunction (five-word test) (Table 2). For formal diagnosis in specialist practice, a more detailed clinical assessment, including neuropsychological testing, may be considered. Table 2 provides examples of other tools and their use within the AD continuum. While attributes of a specific tool dictate where it will be most useful, there is no consensus as to which tool is most appropriate for a specific clinical environment or time point along the continuum. In the future, we will certainly see computerized testing reaching clinical practice as well as the general population at large. While batteries will provide standardized and more rapid testing, they have not yet been developed sufficiently to be adopted widely.

**Table 2:** Examples of clinical tools used and when in the disease course they are most useful

	Examples of tools		
	Brief tools for general setting	Neuropsychological testing	Clinical trials
Clinically normal/SCI	CFI [55]		ADCS-PACC^a^ [38]
			FCI [56]
MCI due to AD/prodromal AD	MIS [57]	FCSRT [58]	FCSRT [58]
	AD8 [59]	RBANS [63]	ADCOM^a^ [60]
	GPCOG [61]	CVLT [66]	FCI [56]
	Mini-cog [62]		RBANS [63]
	Five-word test [64, 65]		
	CFI [55]		
	MoCA [67]		
	ACE III [68]		
	MMSE [69]		
Dementia	MMSE [69]	ADAS-Cog [70]	ADAS-Cog [70]
	ACE III [68]	RBANS [63]	CDR [71]
			RBANS [63]
			ADCS-ADL [53]
	SIB [72]		SIB [72]

^a^Composite tools—comprised of select items from existing scales

*ACE III* Addenbrooke’s Cognitive Examination-III, *AD* Alzheimer’s disease, *AD8* Eight-item Informant Interview to Differentiate Aging and Dementia, *ADAS-Cog* Alzheimer’s Disease Assessment Scale—cognitive subscale, *ADCOM* AD composite, *ADCS* Alzheimer’s Disease Cooperative Study, *ADL* activities of daily living, *CDR* Clinical Dementia Rating, *CFI* Cognitive Function Instrument, *CVLT* California Verbal Learning Test, *FCI* Financial Capacity Instrument, *FCSRT* Free and Cued Selective Reminding Test, *GPCOG* General Practitioner Assessment of Cognition, *MCI* mild cognitive impairment, *MIS* Memory Impairment Screen, *MMSE* Mini-Mental State Examination, *MoCA* Montreal Cognitive Assessment, *PACC* Preclinical Alzheimer Cognitive Composite, *RBANS* Repeatable Battery for the Assessment of Neuropsychological Status, *SIB* Severe Impairment Battery, *SCI* subjective clinical impairment

In recent years, the focus in tool development has been on more sensitive cognitive measures that are able to detect subtle cognitive and functional impairment, to lower the threshold at which clinical disease can be detected as well as to detect smaller changes in cognition/function, to monitor disease progression along the continuum or therapeutic responses to interventions more accurately. Computerized tests that can be readily applied as screening tools in the primary care setting are also being introduced.

One key purpose of clinical assessment is to be able to predict the temporal path of AD at the individual level; for example, if and when MCI due to AD or prodromal AD will progress to AD dementia. Impairment in episodic memory (i.e., the ability to learn and retain new information) is seen most commonly in individuals who subsequently progress to a diagnosis of AD dementia [42, 54]. Based on this, tools that assess episodic memory, both immediate and delayed recall, are valuable. Decline in executive functions may also flag incident AD dementia; by contrast, change in information processing speed/attention seems less informative [54].

## The future of AD management

In the development of therapeutic interventions, there has generally been a shift in focus from AD dementia to MCI due to AD/prodromal AD and earlier; there is a need for a parallel shift in the diagnostic domain, beyond the research environment, to encourage lifestyle modifications and participation in clinical trials. An appreciation of the disease continuum engenders an awareness of our need to consider both diagnosis and therapies in a similar manner, along a continuum. That is, an individual treatment or management option may be most appropriate at a defined stage along the continuum, but its use will likely extend beyond this stage, and there will be overlap among the various treatment and management options such that more than one may be appropriate at any point on the continuum. This will be particularly relevant as new DMTs with different mechanisms of action become available.

With DMTs, treatment earlier in the disease continuum will likely be required to achieve more disease modification and maximize the opportunity of having an effect through slowing decline. If and when DMTs are approved for use, biomarker testing is likely to be central for early and more accurate diagnosis as well as for monitoring treatment effect. We can envision that biomarker findings will influence both initiation and termination of a specific treatment and may help determine whether combination therapies are appropriate and effective at the individual patient level. In particular, amyloid biomarkers will likely be necessary to identify candidates for anti-amyloid interventions at the predementia stages of AD.

The goal of managing any disease is primary prevention and, ultimately, we anticipate blood-based assays of amyloid dysregulation or other changes that will enable primary prevention studies. We can envision eventual management of such indicators of risk of amyloid accumulation with secretase inhibitors, other drug therapies, and lifestyle management to minimize reversible contributors to disease.

## Conclusions/discussion

While we have traditionally described AD in terms of clinically apparent stages, we now have enough understanding of the disease course from pathophysiological, biomarker, and clinical perspectives to appreciate the need to consider AD as a continuum. That is, a process in which pathophysiological changes accumulate and eventually culminate in clinically apparent disease, which then progresses with gradual worsening of cognitive and functional abilities; there are no firm boundaries between the various clinical stages. Based on what we know today, we have endeavored to describe important characteristics along the AD continuum from disease inception to advanced clinical disease. As noted throughout, there is considerable individual variation along the continuum.

As efforts to detect disease earlier in the continuum and to assess disease more accurately continue, use of biomarkers is becoming a central facet of accurate diagnosis, and the presence or absence of the disease may be determined using biomarkers of AD pathology. While routine biomarker assessment of asymptomatic individuals is not currently justified, use of well-defined biomarkers may provide useful prognostic information in individuals with early subjective cognitive decline, as well as help to establish therapeutic goals of clinical care.

Support for the disease continuum concept is growing, yet how this will be successfully integrated into clinical practice is not yet clear. For example, even with the currently available biomarkers and clinical tools, identifying individuals who will progress along the AD spectrum, as well as the trajectory of decline, is still fraught with challenges. Indeed, patients and caregivers only raise concerns with their physician when cognitive deficits are obvious, and this likely correlates with later stages of the development of AD neuropathology. In the future, it is hoped that, with better understanding of the AD continuum, earlier detection will lead to both early and accurate diagnosis and intervention. By 2025 it is hoped that effective DMTs will be approved, and biomarker assessment, together with the employment of more sensitive clinical tools, will become the standard of care. Ultimately, it is predicted that we may need to perform a detailed biomarker assessment for individualized risk prediction to ensure treatments reach individuals at the appropriate time to maximize effects.

## Abbreviations

Aβ: Amyloid beta
AD: Alzheimer’s disease
ADL: Activities of daily living
APOE4: Apolipoprotein protein epsilon 4
APP: Amyloid beta precursor protein
A/T/N: Amyloid, tau, and neurodegeneration
CSF: Cerebrospinal fluid
DMT: Disease-modifying therapy
MCI: Mild cognitive impairment
MMSE: Mini-Mental State Examination
NFT: Neurofibrillary tangle
PART: Primary age-related tauopathy
PET: Positron emission tomography
